# Attitude of Jordanian Health Care Workers Toward Surrogacy

**Published:** 2020-03

**Authors:** Rami Saadeh, Nancy Abdulrahim, Mahmoud Alfaqih, Yousef Khader

**Affiliations:** 1 Department of Public Health and Community Medicine, Faculty of Medicine, Jordan University of Science and Technology, Irbid, Jordan; 2 Jordanian Ministry of Health, Amman, Jordan; 3 Department of Physiology and Biochemistry, Faculty of Medicine, Jordan University of Science and Technology, Irbid, Jordan

**Keywords:** Jordan, Attitude, Healthcare Workers, Surrogacy

## Abstract

**Objective:** To assess the attitude of Jordanian health care workers toward surrogacy.

**Materials and methods:** Three municipalities in Jordan were randomly selected, one from each region: north, south and central of Jordan. A total of: four public hospitals, three private hospitals, one university hospital, 40 health centers and 40 private clinics were included in the study. Healthcare workers in the selected facilities were randomly approached using a self– administered questionnaire to collect data. Distributions of attitude by gender, job title, and physician’s specialty were used to describe participants’ attitude toward surrogacy.

**Results:** Responses of 382 participants were reported, of whom, 230 (60.2%) were females. Three in every four participants didn’t support legalizing surrogacy in Jordan. Majority reported negative attitude toward commercial surrogacy (85.1%) and noncommercial surrogacy (76.4%). Religious considerations were the main reason (71.1%) for the attitude toward surrogacy. Most items describing attitude toward surrogacy were significantly distributed across different job titles: nurses, medical doctors, and other healthcare workers (p < 0.05).

**Conclusion:** Negative attitude among health care workers toward surrogacy was mainly driven by religious beliefs. However, there are core cultural changes in the community which might alter the attitude toward surrogacy in the future.

## Introduction

Surrogacy, or surrogate motherhood, is a method of assisted reproduction whereby a woman agrees to become pregnant for the purpose of gestating and giving birth to a child who will be raised by others (1).

Gestational surrogacy, which is to gestates a gamete produced through in vitro fertilization (IVF) of a commissioning couple (2), was reported 25 years ago (3). Since then, gestational surrogacy became a viable treatment for couples who cannot conceive because of a nonfunctional uterus, encounter high risks associated with pregnancy, or experienced failure to conceive naturally or through using other treatment options (4-6).

However, due to its complicated ethical issues, gestational surrogacy is outlawed in many countries worldwide. While banned in many countries, surrogacy is legally practiced but inadequately regulated in some others, driving the need for a better level of regulation (6, 7).

Laws and regulations that prohibit or allow the practice of surrogate pregnancy consider many factors. For example, regulations governing this practice often differentiate between two types of surrogacy: 1) traditional surrogacy where the surrogate mother’s own eggs are fertilized via intrauterine insemination by sperm of the future father and 2) gestational surrogacy where the surrogate mother carries the egg of the intended mother. The latter one uses in vitro fertilization (IVF) to make of the intended mothers’ egg and the intended father’s sperm to produce the embryo (6-9). Regulations also differentiate between commercial surrogacy where the surrogate mother is paid for pregnancy and altruistic surrogacy where the surrogate mother is reimbursed for the expenses of pregnancy (7).

Attitude toward surrogacy varies worldwide and is influenced by religion, gender, age, socioeconomic status, and knowledge about the procedure. Studies reported conflicting findings about attitude toward surrogacy. For example, permissive attitude toward several new reproductive technologies such as IVF among women was reported in Sweden except for surrogacy (10). Whereas in Germany, comparable rates of approval and disapproval toward surrogacy were reported (11). In Turkey, less acceptance of surrogacy was noted (12). However, in Japan, it is suggested that many factors, including socioeconomic status, affect a person’s ability to clearly express an opinion about surrogacy (13). In contrast, Iranian infertile women believed that surrogacy is better than adoption or not having children; reflecting a positive attitude toward surrogacy in general (14).

It is suggested that acceptance of surrogacy might be affected by gender. In Canada, for example, men were significantly more willing to consider using surrogacy in one study (15). Another in Germany, men and women were not significantly different (11). However, it was reported in Japan that gender differences toward surrogacy could exist but was rather affected by information provided about the procedure (16).

Furthermore, it is suggested that religion has an important impact on people’s attitude toward surrogacy. Because surrogacy is forbidden in Islam, Islamic countries consider surrogacy inadmissible, beholding the religious view and not necessary the law (17, 18). Comparatively, the Catholic Church is strongly against all forms of assisted reproduction, particularly those associated with third party assistance or surrogacy. In Egypt, Christians’ beliefs concerning assisted reproduction were found to be identical to Muslims (19, 20). However, surrogacy is not forbidden in the Jewish religion because the value of having a child in this community is viewed to outweigh any ethical concerns counted by reproductive technologies (18). This attitude made Jews one of the leading communities in the research and development of new reproductive technologies.

Attitude toward the use of a third party-assisted reproduction in Jordan was investigated in one study reporting views of Jordanian medical students. The study indicated a general reluctance toward accepting the concept of surrogate pregnancy, mainly driven by religious beliefs (21). However, the attitude toward surrogacy among health care workers (HCWs) in Jordan was never examined before. This study clarified the level of acceptance, and therefore, what they may stand for in its regulation. 

## Materials and methods

***Study design: ***A descriptive, cross-sectional study design using a self–administered questionnaire was utilized to collect data between May and August 2019. Cities in Jordan were clustered into the three main regions of Jordan: north, center, and south. One city from each region was randomly selected for data collection. The selected cities were Irbid in the north, Amman in the center, and Karak in the south. There are no predetermined groups of health care workers that were selected for the study but rather participants were invited randomly to participate in the study. Researcher who collected data was trained to ensure that both sampling and data collection were done anonymously and randomly.

**Figure 1:**
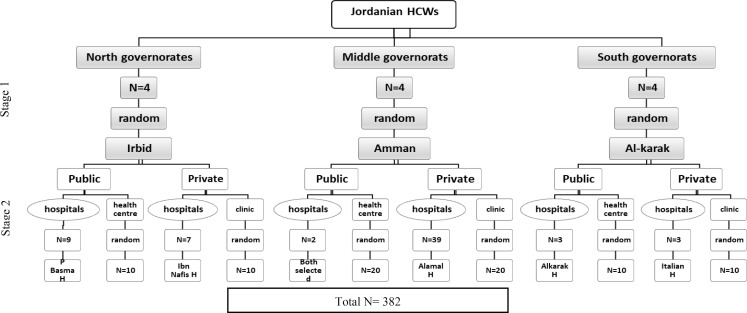
Illustration of the Sampling Method Used

***Study settings: ***This study was carried out at health care facilities in the Municipality of Amman, Irbid and Alkarak, Jordan. Four public hospitals, one university hospital and three private hospital located in north, middle and south of Jordan were randomly selected. The 2 public hospitals selected in Amman were: Al Bashir hospital which is the largest public hospital in Jordan and Al Totangi hospital. Both have departments of obstetrics and gynecology. The university hospital was Jordan university hospital which is the oldest teaching hospital in Jordan. The private hospital was Al Amal maternity hospital which contains infertility treatment center and provides reproductive services for over 20 years. Two hospitals were randomly selected in north of Jordan, one public (Princess Basma Hospital) and one private (Ibn Al–Nafis hospital) located in Irbid. Two hospitals were randomly selected in south of Jordan, one public (Alkarak Hospital) and one private (Italian Hospital). In addition, 40 private clinics and 40 health centers were further included to increase the diversity of participants selected (Figure 1).

***Sample size calculation: ***According to the 2018 annual statistical report for the Ministry of Health, the total number of HCWs in private sector in Jordan is 44261; the total number of those working in the public sector is 19307, and 3151 working in university hospitals. The total number of the target population is 66719. Using Epi Info 7 software, the required sample for a population survey with 95 confidence interval and 5 margin of error was 382. Although a 10 attrition rate, which is equal to 39 individuals, should have been added to the required sample, only 382 responses could be sampled because of the high rate no response. Among 500 health care workers approached, only 382 agreed to participate, leading to a response rate of 76.4.

***Data collection instruments: ***The questionnaire was developed based on previous studies that examined the attitude of HCWs who were involved with reproduction services during their medical career (17, 22). However, some items were modified to meet the objectives of this study, and therefore, was validated to ensure that the objectivity of questions were valid. Face and content validation were achieved by the revision of 10 experienced physicians working in the field of obstetrics and reproductive medicine and two epidemiologists who assisted in phrasing the questions. The survey was pilot tested on 20 health care professionals to obtain their input. Their feedback was used to adjust for minor revisions, and responses were used to assess the internal consistency of the questionnaire using Cronbach's alpha test of reliability, which was 0.78; providing a good reliability.

***Study variables:*** The survey was consisted of 2 sections containing 15 items: socio-demographic information (7 items) and attitude toward surrogacy (8 items). The respondents were informed to rate each statement about their beliefs either as “Agree”, Cannot decide”, or “Disagree”.

***Statistical analysis: ***Categorical variables were expressed as frequencies with percentages and continuous variables as mean ± standard deviation. Categorical variables were compared using the chi-squared test. All analyses were performed by the two-sided method using IBM Statistical Package for Social Sciences (SPSS) version 23, and the P-value of < 0.05 was set as statistically signiﬁcant.

***Ethical Considerations: ***The protocol of the study was approved by the institutional review board (IRB) of Jordan University of Science and Technology prior to collection of data. Participants were informed about their right to refuse participating in the study and withdraw at any time. The data was kept confidential and no identifying information was obtained from participants.

## Results

***Socio-demographic characteristics: ***A total of 382 forms were completed, of whom 230 (60.2) were females. Age ranged between 20 and 60. The Mean age was 35.3 years (± 9.9). Participants had a wide range of work experience; ranging from one to thirty years, with mean of 10.5 (± 8.25) years. Majority of respondents 365 (95.5) were Muslims, while Christians accounted for 3, and the rest selected “other” religions (0.6). Most of respondents were working in the public sector (55.7), and 126 (33) were working in the private sector. Married participants were 267 (69.9), 95 (24.9) were single, 16 (4.2) were separated and only 4 (1) were widows. Medical doctors (including general practitioners, residents and specialist) composed 41.6 of respondents. Table 1 summarizes characteristics of study participants.

**Table 1:** Characteristics of Study Participants

**Characteristics**		**Frequency**	**%**
Gender	Male	152	39.8
	Female	230	60.2
	Total	382	100.0
Religion	Islam	365	95.5
	Christianity	11	3.0
	Other	2	0.6
	Didn’t respond	4	0.9
	Total	382	100.0
Place of residence	City	313	81.9
	Village	67	17.5
	Didn’t respond	2	0.6
	Total	382	100.0
Place of work	Health center	38	9.9
	Public hospital	175	45.8
	Private hospital	95	24.9
	University hospital	31	8.1
	Private clinic	31	8.1
	Didn’t respond	12	2.2
	Total	370	100.0
Marital status	Single	95	24.9
	Married	267	69.9
	Separated	16	4.2
	Widow	4	1.0
	Total	382	100.0
Specialization	Nurse	123	32.2
	Midwifery	16	4.2
	General practitioner	49	12.8
	Resident	44	11.5
	Specialist	68	17.8
	Technician	22	5.8
	Dentist	13	3.4
	Pharmacist	17	4.5
	Didn’t respond	30	8.3
	Total	382	100.0
What is your specialty (both residents & specialists)?	Gynecologist	55	14.4
	Pediatrician	20	5.2
	Other specialty	75	19.6
	Total	150	39.3

***Participants knowledge and attitude toward surrogacy: ***Most participants reported negative attitude towards surrogacy whether it is done for non–commercially, which is free of charge (n = 292 or 76.4), or commercially (n = 325 or 85.1). The main reason behind this belief was religious motivation (n = 274 or 71.7). Although 241 (63.1) know the difference between two types of surrogacy, the majority (n = 311 or 81.4) didn’t support organic (gestational) surrogacy nor genetic (traditional) surrogacy. However, few were supportive to organic surrogacy (n = 37 or 9.7) or genetic surrogacy (n = 4 or 1). Some (n = 24 or 6.3) supported both if needed (Table 2). 

**Table 2:** Participants’ Attitude toward Surrogacy

**Attitude**	**Response**	**Frequency**	**%**
I support surrogacy if its free of charge (Noncommercial)	No	292	76.4
	Cannot decide	48	12.6
	Yes	39	10.2
	Didn’t respond	3	0.8
	Total	382	100
I support the commercial Surrogacy	No	325	85.1
	Cannot decide	34	8.9
	Yes	20	5.2
	Didn’t respond	3	0.8
	Total	382	100
Do you know the difference between genetic and organic Surrogacy?	No	137	35.9
	Yes	241	63.1
	Didn’t respond	4	1
	Total	382	100
Do you Support the genetic (Egg and womb) or organic (womb only) Surrogacy?	I don’t support any	311	81.4
	Organic	37	9.7
	Genetic	4	1
	I support both if needed	24	6.3
	Didn’t respond	6	1.6
	Total	382	100
In the event of Surrogacy, do you prefer:	Strange donor	157	41.1
	Friends	22	5.8
	Relatives	65	17
	Didn’t respond	138	36.1
	Total	382	100
The reason behind the attitude towards surrogacy motivated by:	Religious	274	71.7
	Social	32	8.4
	Legal	9	2.4
	Other	49	12.8
	Didn’t respond	18	4.7
	Total	382	100
Is it possible to be more positive toward surrogacy if you know that it's the only way to have children?	No	207	54.2
	Cannot decide	108	28.3
	Yes	58	15.2
	Didn’t respond	9	2.4
	Total	382	100
Do you support legalizing surrogacy in Jordan?	No	286	74.9
	Cannot decide	52	13.6
	Yes	40	10.5
	Didn’t respond	4	1
	Total	382	100

The distribution of participants’ attitude toward surrogacy based on gender, job title, and physicians’ specialty are shown in Tables 3, 4, 5 respectively (Table 3, 4, 5). As shown in Table 3, there was no statistically significant difference between genders in most items of attitude toward surrogacy. 

It’s interesting that there was a statistically significant relationship between job titles and most items of attitude toward surrogacy (p < 0.05) (Table 4). Considering the total number of HCWs under each job title, medical doctors were the most supportive of surrogacy (18) among others, and nurses/midwifes were the least supportive (2).

Moreover, the attitude of nurses/midwifes toward surrogacy was largely driven by religion (85), and absolutely absent by any legal considerations (0). It’s interesting that there was no significant difference among HCWs regarding their knowledge about the difference between genetic and organic surrogacy (p = 0.416).

There was no significant difference between specialists, whether they are gynecologists, pediatricians or other specialties, regarding their attitude toward surrogacy (Table 5). 

## Discussion

This study focused on assessing the attitude of HCWs toward surrogacy. The importance of this assessment emerged from the crucial role of HCWs in ensuring the applicability and/or the acceptability of these practices in the Jordanian communities, in addition to their ability to advocate for policies that support or refute its permittance. 

The ethical, legal, and religious dilemmas associated with surrogacy resulted in conflicting opinions toward it (7, 9, 22).

**Table 3:** Attitude toward surrogacy distributed by gender

**Attitude toward surrogacy**			**Gender**		**Total** **N (%)**	**P-value** *
			**Male** **N (%)**	**Female** **N (%)**		
Do you support legalizing surrogacy in Jordan?	No	106 (70.2)		180 (79.3)	286 (75.7)	0.075
	Cannot decide	23 (15.2)		29 (12.8)	52 (13.8)	
	Yes	22 (14.6)		18 (7.9)	40 (10.6)	
I support the commercial Surrogacy	No	123 (81.5)		202 (88.6)	325 (85.8)	0.110
	Cannot decide	19 (12.6)		15 (6.6)	34 (9.0)	
	Yes	9 (6.0)		11 (4.8)	20 (5.3)	
I support surrogacy if its free of charge (Noncommercial)	No	111 (73.0)		181 (79.7)	292 (77.0)	0.243
	Cannot decide	21 (13.8)		27 (11.9)	48 (12.7)	
	Yes	20 (13.2)		19 (8.4)	39 (10.3)	
Do you know the difference between genetic and organic Surrogacy?	No	49 (32.5)		88 (38.8)	137 (36.2)	0.211
	Yes	102 (67.5)		139 (61.2)	241 (63.8)	
Do you Support the genetic (Egg and womb) or organic (womb only) Surrogacy?	I don't support any of them	116 (77.3)		195 (86.3)	311 (82.7)	0.131
	Organic	18 (12.0)		19 (8.4)	37 (9.8)	
	Genetic	2 (1.3)		2 (0.9)	4 (1.1)	
	I support both if needed	14 (9.3)		10 (4.4)	24 (6.4)	
In the event of Surrogacy, do you prefer:	Strange donor	56 (61.5)		101 (66.0)	157 (64.3)	0.656
	Friends	10 (11.0)		12 (7.8)	22 (9.0)	
	Relatives	25 (27.5)		40 (26.1)	65 (26.6)	
The reason behind the attitude towards surrogacy motivated by:	Religious	100 (68.5)		174 (79.8)	274 (75.3)	0.007
	Social	16 (11.0)		16 (7.3)	32 (8.8)	
	Legal	8 (5.5)		1 (0.5)	9 (2.5)	
	Other	22 (15.1)		27 (12.4)	49 (13.5)	
Is it possible to be more positive toward surrogacy if you know that it's the only way to have children?	No	77 (51.7)		130 (58.0)	207 (55.5)	0.073
	Cannot decide	41 (27.5)		67 (29.9)	108 (29.0)	
	Yes	31 (20.8)		27 (12.1)	58 (15.5)	

* Chi-Square test

**Table 4:** Attitude toward surrogacy distributed by Job title

**Attitude toward surrogacy**		**Gender**			**Total** **N (%)**	**P-value** *
		**Nurse or ** **midwifery** **N (%)**	**Medical ** **doctor** **N (%)**	**Other health ** **professionals** **N (%)**		
Do you support legalizing surrogacy in Jordan?	No	119 (85.6)	120 (67.8)	28 (84.8)	267 (76.5)	< 0.001
	Cannot decide	17 (12.2)	25 (14.1)	4 (12.1)	46 (13.2)	
	Yes	3 (2.2)	32 (18.1)	1 (3.0)	36 (10.3)	
I support the commercial Surrogacy	No	128 (91.4)	145 (81.5)	30 (90.9)	303 (86.3)	0.005
	Cannot decide	12 (8.6)	17 (9.6)	1 (3.0)	30 (8.5)	
	Yes	0 (0.0)	16 (9.0)	2 (6.1)	18 (5.1)	
I support surrogacy if its free of charge (Noncommercial)	No	119 (85.6)	126 (70.8)	28 (84.8)	273 (78.0)	< 0.001
	Cannot decide	18 (12.9)	22 (12.4)	3 (9.1)	43 (12.3)	
	Yes	2 (1.4)	30 (16.9)	2 (6.1)	34 (9.7)	
Do you know the difference between genetic and organic Surrogacy?	No	55 (39.6)	59 (33.3)	10 (30.3)	124 (35.5)	0.416
	Yes	84 (60.4)	118 (66.7)	23 (69.7)	225 (64.5)	
Do you Support the genetic (Egg and womb) or organic (womb only) Surrogacy?	I don't support any of them	131 (94.2)	129 (73.7)	29 (87.9)	289 (83.3)	< 0.001
	Organic	4 (2.9)	25 (14.3)	3 (9.1)	32 (9.2)	
	Genetic	1 (0.7)	2 (1.1)	1 (3.0)	4 (1.2)	
	I support both if needed	3 (2.2)	19 (10.9)	0 (0)	22 (6.3)	
In the event of Surrogacy, do you prefer:	Strange donor	54 (60.7)	75 (64.7)	16 (76.2)	145 (64.2)	0.757
	Friends	9 (10.1)	11 (9.5)	1 (4.8)	21 (9.3)	
	Relatives	26 (29.2)	30 (25.9)	4 (19.0)	60 (26.5)	
The reason behind the attitude towards surrogacy motivated by:	Religious	113 (85.0)	115 (67.3)	26 (81.3)	254 (75.6)	0.008
	Social	5 (3.8)	20 (11.7)	2 (6.3)	27 (8.0)	
	Legal	0 (0.0)	8 (4.7)	1 (3.1)	9 (2.7)	
	Other	15 (11.3)	28 (16.4)	3 (9.4)	46 (13.7)	
Is it possible to be more positive toward surrogacy if you know that it's the only way to have children?	No	87 (63.5)	87 (50.0)	23 (69.7)	197 (57.3)	0.002
	Cannot decide	40 (29.2)	48 (27.6)	8 (24.2)	96 (27.9)	
	Yes	10 (7.3)	39 (22.4)	2 (6.1)	51 (14.8)	

* Chi-Square test

While some communities support the practice of surrogacy to assist millions of infertile couples who lost hope to have children, many others consider it morally unacceptable and unjustified technology (7, 9, 19, 20, 22-24). In this study, results show that about 24 of participants were either positive or neutral toward legalization surrogacy in Jordan. This is different than findings of a recent study of Jordanian medical and paramedical students in which only 5.5 of them supported it and 7.6 were neutral (21). This level of support was close to a Turkish study where only 15.1 of infertile women approved using gestational surrogacy (12). However, other countries showed differently. In a Japanese study, nearly 50 of the respondents initially supported gestational surrogacy. Later, disapproval rate increased after reading a brochure explaining the merits and risks of the technology (13). More information could result into more cautious attitudes. Iran is the only Muslim country that permits surrogacy, both altruistic and commercial, which are legitimized by law and religious authorities.

An Iranian study assessing citizens’ acceptability to surrogacy showed that many Iranian preferred surrogacies when needed (25).

Some communities are distinguished by their high level of acceptance to surrogacy. For example: 1) a Swedish study demonstrated that 63 of physicians were positive or neutral toward altruistic surrogacy being introduced in Sweden (26), 2) a Romanian study showed that 78 of physicians had high acceptance of ARTs including surrogacy (27), and 3) a British study found that 72 of medical students considered surrogacy as an acceptable form of assisted reproduction (28).

**Table 5:** Attitude toward surrogacy distributed by physicians’ specialty

**Attitude toward surrogacy**		**Gender**			**Total** **N (%)**	**P-value** *
		**Gynecologists** **N (%)**	**Pediatricians** **N (%)**	**Other ** **specialties** **N (%)**		
Do you support legalizing surrogacy in Jordan?	No	40 (75.5)	17 (85.0)	50 (66.7)	107 (72.3)	0.471
	Cannot decide	5 (9.4)	2 (10.0)	11 (14.7)	18 (12.2)	
	Yes	8 (15.1)	1 (5.0)	14 (18.7)	23 (15.5)	
I support the commercial Surrogacy	No	42 (79.2)	18 (90.0)	60 (80.0)	120 (81.1)	0.875
	Cannot decide	6 (11.3)	1 (5.0)	8 (10.7)	15 (10.1)	
	Yes	5 (9.4)	1 (5.0)	7 (9.3)	13 (8.8)	
I support surrogacy if its free of charge (Noncommercial)	No	37 (68.5)	15 (75.0)	52 (69.3)	104 (69.8)	0.967
	Cannot decide	9 (16.7)	3 (15.0)	11 (14.7)	23 (15.4)	
	Yes	8 (14.8)	2 (10.0)	12 (16.0)	22 (14.8)	
Do you know the difference between genetic and organic Surrogacy?	No	15 (27.8)	7 (35.0)	33 (44.6)	55 (37.2)	0.148
	Yes	39 (72.2)	13 (65.0)	41 (55.4)	93 (62.8)	
Do you Support the genetic (Egg and womb) or organic (womb only) Surrogacy?	I don't support any of them	41 (77.4)	17 (85.0)	58 (78.4)	116 (78.9)	0.992
	Organic	7 (13.2)	2 (10.0)	10 (13.5)	19 (12.9)	
	Genetic	1 (1.9)	0 (0.0)	1 (1.4)	2 (1.4)	
	I support both if needed	4 (7.5)	1 (5.0)	5 (6.8)	10 (6.8)	
In the event of Surrogacy, do you prefer:	Strange donor	20 (58.8)	11 (61.1)	40 (72.7)	71 (66.4)	0.240
	Friends	2 (5.9)	3 (16.7)	2 (3.6)	7 (6.5)	
	Relatives	12 (35.3)	4 (22.2)	13 (23.6)	29 (27.1)	
The reason behind the attitude towards surrogacy motivated by:	Religious	36 (69.2)	16 (84.2)	50 (67.6)	102 (70.3)	0.436
	Social	9 (17.3)	0 (0.0)	8 (10.8)	17 (11.7)	
	Legal	1 (1.9)	1 (5.3)	3 (4.1)	5 (3.4)	
	Other	6 (11.5)	2 (10.5)	13 (17.6)	21 (14.5)	
Is it possible to be more positive toward surrogacy if you know that it's the only way to have children?	No	28 (54.9)	11 (55.0)	38 (50.7)	77 (52.7)	0.892
	Cannot decide	14 (27.5)	7 (35.0)	23 (30.7)	44 (30.1)	
	Yes	9 (17.6)	2 (10.0)	14 (18.7)	25 (17.1)	

* Chi-Square test

Religion seems to play an important role in determining the attitude toward this and similar issues; since 71.7 of participants referred to religion as the source of their attitude (20, 29). A comparable trend was reported by another Jordanian study showing that religion was a fundamental determinant of attitude toward surrogacy (21). The preservation of lineage, the exclusion of third parties in reproduction, the upholding of the child’s rights, and the protection from the negative effects of surrogacy are the major reasoning in prohibiting surrogacy in Muslim Sunni community (20, 29). One of the fundamental principles in Sunni Islam is protecting the family lineage. Therefore, all forms of surrogacy are forbidden, including the establishment of sperm or egg banks; reasoned by the consequences of this practices which is the destabilization of the communities through threatening the existence of families (20, 30).

Findings of this study illustrated that medical doctors were the most supportive to surrogacy than other HCWs and nurses were the least supportive (p < 0.05); Table 3 and 4. 

Generally, medical doctors are more aware of the cons and pros of these modern medical techniques, and the sustenance of their career relies on such and similar procedures; two possible explanations for the difference between doctors and others.

There was also a slight difference between men and women regarding their attitude toward surrogacy that was not statistically significant, except for the reasons behind their views on surrogacy (p = 0.007). This is consistent with a German study in which women were more inclined to religious reasons than men, while the men were more inclined to social and legal reasons than women (11). The findings of the German study could explain why nurses/midwifery are the least supportive to surrogacy. Females constitute 80 of nurses in this study. At the same time, female’s attitude toward surrogacy is mainly driven by religious reasons, concluding that nurses, who are mainly females, had negative attitude toward surrogacy because of their religious beliefs. However, medical specialties did not show a significant difference in their attitude, probably because of their small sample size or because doctors share the same attitude motivated by their awareness and medical training, regardless of their specialty.

***Limitations:*** The sensitive topics discussed in this study held back many from participating in the study, which resulted in a lower response rate than expected. The small sample size entitles some draw backs, such as: generalizability problem, hindering the existence of possible significant relationships, increasing the probability of error type 2, and decreased power. Another limitation in sampling is that the number of HCWs of different professions who participated in this study was not reflective for their numbers and distribution in hospitals, clinics, and health centers. Thus, proportions based on how personnel are distributed were not considered in sampling and only the total number required for the sample was considered, regardless of the appropriate proportions of each profession.

## Conclusion

There was a general reluctance among HCWs of this study toward accepting surrogacy, which was mainly driven by religious beliefs; indicating the importance of religion in making familial decisions. However, some medical doctors in this study agreed with legalizing surrogacy in Jordan and supported its use. This indicates that changes toward this practice might occur in the future and movements advocating for its permittance could be witnessed among the medical community in the country.
